# The role of lysyl oxidase-like 1 DNA copy number variants in exfoliation glaucoma

**Published:** 2012-12-14

**Authors:** Yutao Liu, Benjamin T. Whigham, Joshua Wheeler, Susan E. I. Williams, Robyn M. Rautenbach, Ari Ziskind, Michele Ramsay, Trevor R. Carmichael, Allison E. Ashley-Koch, R. Rand Allingham, Michael A. Hauser

**Affiliations:** 1 Center for Human Genetics, Department of Medicine, Duke University Medical Center, Durham, NC; 2 Department of Ophthalmology, Duke University Medical Center, Durham, NC; 3 Division of Ophthalmology, Department of Neurosciences, University of the Witwatersrand, Johannesburg, South Africa; 4 Division of Ophthalmology, Department of Surgical Sciences, Stellenbosch University, Stellenbosch, South Africa; 5 Division of Human Genetics, NHLS and School of Pathology, University of Witwatersrand, Johannesburg, South Africa

## Abstract

**Purpose:**

To investigate whether DNA copy number variants (CNVs) in the lysyl oxidase-like 1 (*LOXL1*) gene are associated with exfoliation glaucoma (XFG) in black South Africans.

**Methods:**

Black South African subjects with XFG and age-matched unaffected controls were recruited from the St. John Eye Hospital in Soweto (Johannesburg, South Africa) and East London Hospital Complex (Eastern Cape, South Africa) using standard clinical examination techniques. A customized array comparative genomic hybridization (aCGH) from Roche NimbleGen was designed to cover a 1.5 million base genomic region centered on the *LOXL1* gene on chromosome 15. Twenty selected XFG cases were examined using this custom aCGH to identify common CNVs in the *LOXL1* gene. The potential DNA copy number variants identified from aCGH were further validated using TaqMan probe-based CNV real-time PCR in a data set containing 91 XFG cases and 52 controls. The frequencies of CNVs in the *LOXL1* region were compared between the XFG cases and the controls using Fisher's exact test.

**Results:**

Several DNA CNV variants were identified in the *LOXL1* genomic region using aCGH in the selected XFG cases. However, we were unable to validate these candidate CNVs using real-time PCR-based TaqMan CNV assays. There was no significant difference in the frequency of the DNA copy number variants in the *LOXL1* region between the XFG cases and the controls.

**Conclusions:**

This represents the first DNA CNV study of *LOXL1* in the black South African population with XFG. Our study did not identify any significant DNA copy number alterations in the genomic region containing the *LOXL1* gene. This suggests that other as yet unknown causal variants of *LOXL1* or variants in other genes in linkage disequilibrium with the *LOXL1* locus contribute to the genetic risk of XFG in black South Africans.

## Introduction

Glaucoma is characterized by progressive loss of retinal ganglion cells, optic nerve cupping, and visual field loss [1]. Glaucoma is the second most common cause of vision loss worldwide [2,3]. Exfoliation glaucoma (XFG) is the most common identifiable secondary cause of open-angle glaucoma [4-6]. XFG is a direct result of exfoliation syndrome (XFS), a systemic condition characterized by pathological deposits of microfibrillar material within the eye and other non-ocular tissues [5-8]. Although XFG is uncommon in African Americans and virtually non-existent in West Africans [9-11], it is relatively common in black South Africans and accounts for approximately 16%–20% of glaucoma in that population [12,13]. This ancestral population can be divided into distinct linguistic groups, but they are closely related to each other genetically [14]. Recently, we reported the strong association of coding variants in the lysyl oxidase-like 1 (*LOXL1*) gene with XFG in this population [15,16]. Interestingly, the major allele of rs3825942 that confers increased risk of XFG in all non-African populations is associated with reduced risk in South Africans [4,15-17]. This finding, in addition to the fact that the two associated variants, R141L (rs1048661) and G153D (rs3825942) [17-21], have been shown to have no effect on the amine oxidase activity of LOXL1 protein, suggests that these *LOXL1* variants tag the true functional variants [22]. These functional variants remain unknown [4], but may exert an effect through disruption of *LOXL1* regulation [4,8,23].

DNA copy number variants (CNVs) have been shown to be responsible for phenotypic variations [24] as well as human genetic disorders, including primary open-angle glaucoma [25,26], normal tension glaucoma [27], and age-related macular degeneration [28-31]. CNVs can change the copy number of entire genes, or may duplicate or delete regulatory elements that affect the expression level of one or more genes [24]. Given the reported alteration of *LOXL1* expression in XFS and XFG [8,20,32,33], it is important to examine the *LOXL1* region for potential disease-associated CNV changes, studies that have not been performed to date in XFS or XFG.

To identify potential CNVs encompassing the *LOXL1* gene, we applied a two-stage approach. First, we designed a custom comparative genomic hybridization array targeting the *LOXL1* locus in a set of 20 XFG cases. Second, we used TaqMan-based real-time PCR in 91 XFG cases and 52 controls to validate candidate CNV changes. This is the first report to examine the roles of *LOXL1*-related CNVs in XFG, especially in black South African individuals.

## Methods

### Study participants

This study adhered to the tenets of the Declaration of Helsinki. The research protocol was approved by all participating universities including the University of the Witwatersrand Human Research Ethics Committee and Stellenbosch University Health Research Ethics Committee. Black Southern African participants with clinically diagnosed XFG and unaffected control subjects were recruited from the St. John Eye Hospital in Soweto (Johannesburg, South Africa) and East London Hospital Complex (Eastern Cape, South Africa), and have been previously described [15,16]. Written informed consent was obtained from all participants. Ethnic affiliation was established by the home language of participants and that of their parents and grandparents. All participants underwent a standardized detailed ophthalmic examination by an ophthalmologist (S.E.I.W. or R.M.R.). The examination included measurement of intraocular pressure (IOP) by applanation, slit-lamp biomicroscopy, gonioscopy, dilated pupil examination of the lens and fundus, and visual field testing. Subjects with XFG were defined as those with clinical evidence of exfoliation material on the pupil margin, anterior lens surface, and the presence of glaucomatous optic neuropathy and visual field loss. Gender- and ethnicity-matched South African subjects with normal IOP and normal anterior segment and optic nerve examinations were recruited as control subjects. Control subjects were older than 55 years of age to reduce the possibility of misdiagnosis.

### DNA copy number analysis with array comparative genomic hybridization

Genomic DNA was extracted using a salting out procedure from nucleated cells from the venous blood samples from all subjects [34]. A total of 91 XFG cases and 52 controls were included in our study. Twenty XFG cases were selected for Roche NimbleGen (Madison, WI) array CGH analysis using custom designed arrays targeting the genomic region around the *LOXL1* gene. The array CGH was performed as described previously [25]. Briefly, 500 ng high-quality undegraded genomic DNA was labeled with Cy3, and the reference sample from HapMap was labeled with Cy5. The hybridization was done according to the standard procedures recommended by the manufacturer. The arrays were scanned on an Axon4100A scanner (Molecular Devices, Sunnyvale, CA). Scanned TIFF images were processed using NimbleScan 2.6 software. The CNV calls were determined using segMNT algorithm in NimbleScan and were visualized using SignalMap 1.8 from Roche NimbleGen. Briefly, blood was obtained using peripheral venipuncture. The blood was anticoagulated and lysed. Cellular protein was salted out of solution using NaCl. Genomic DNA, still in solution, was removed, precipitated in ethanol, and resuspended in water.

### Copy number variant validation and screening with real-time polymerase chain reaction

To validate the CNVs identified from the array CGH experiments, we selected three candidate CNVs in the *LOXL1* genomic region. We used TaqMan Copy Number Assays from ABI (Applied Biosystems Inc., Carlsbad, CA) on the ABI Prism 7900HT Sequence Detection System for validation and further screening in all XFG cases and controls. A Vic-labeled Copy Number Assay for RNase P was selected as an internal control as it performed in the same reaction with gene-specific assays. Four Copy Number Assays were ordered from ABI. Hs01538855_cn, HS01338991_cn, and HS00942919_cn assays to target the exon 1 coding region, which would be used to validate any CNVs overlapping with the *LOXL1* exon 1. The Hs03894435_cn assay targets intron 6 of the *LOXL1* gene, which could be used to validate any CNVs affecting the *LOXL1* intron 6. Each sample was assayed with four replicates by using 10 ng DNA in each reaction in a 384-well format. The CNV calls were generated with SDS software and CopyCaller from ABI (Applied Biosystems). A known CEPH sample was used as a reference for a copy number of 2. This CEPH sample had been previously confirmed with a single nucleotide polymorphism microarray to contain two copies of candidate CNV regions. To make CNV calls in CopyCaller software, a confidence score of greater than 0.95 was required with four replicates.

## Results

The cases in this study had exfoliation-related open-angle glaucoma [15,16]. Our data set included 91 black South Africans with XFG and 52 black South African controls. The mean age at enrollment for the cases and controls was 75.01±8.98 and 74.5±8.49 years, respectively. The percentage of women in our data set was 41% in the cases and 56% in the controls.

**Figure 1:**
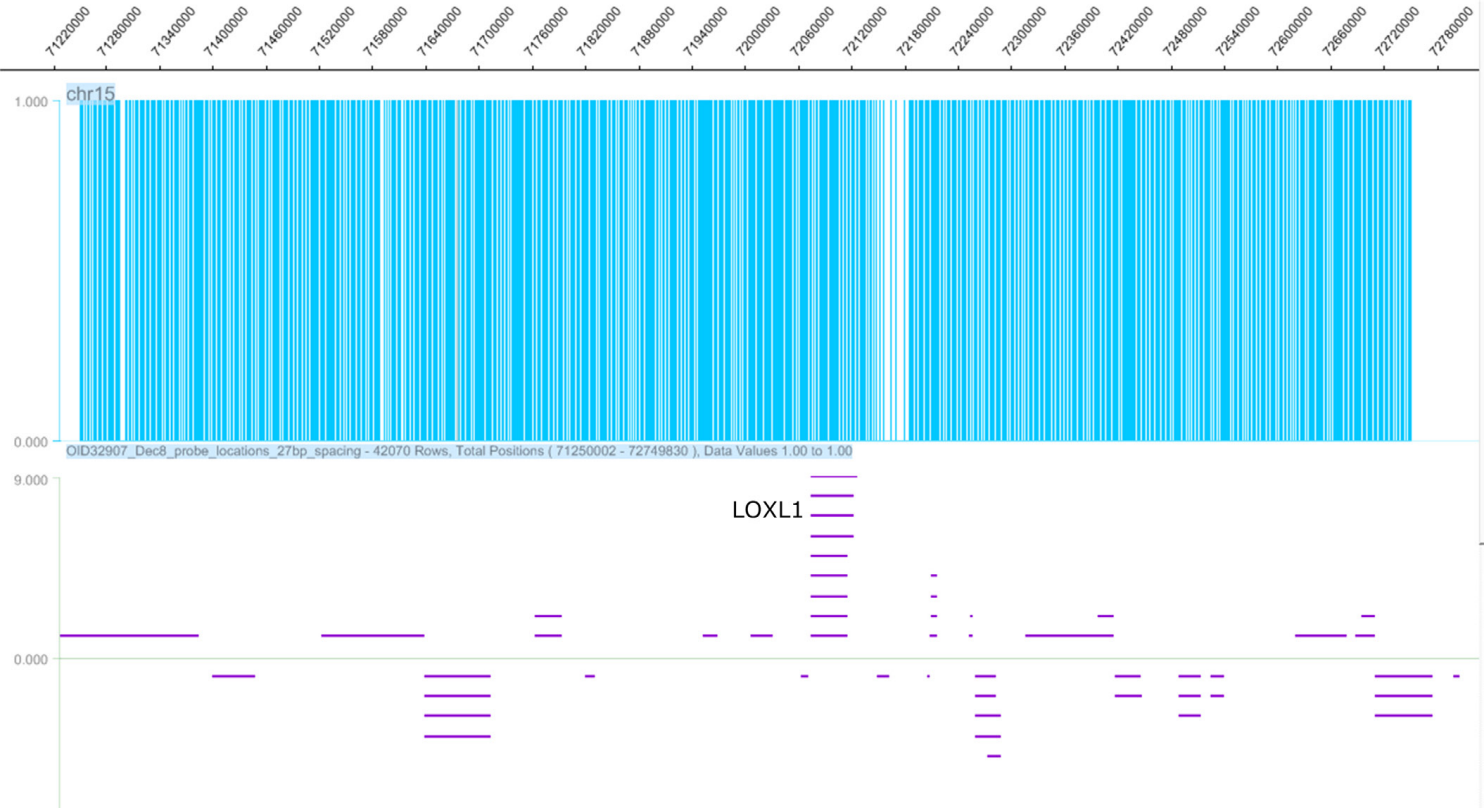
The copy number probe coverage in the genomic region containing the *LOXL1* gene. The top panel with blue lines indicates the probe density, and the bottom panel with purple lines shows the genes in the selected region. The *LOXL1* gene is located in the center of the selected region. The overall region is approximately 1.5 Mb.

**Figure 2:**
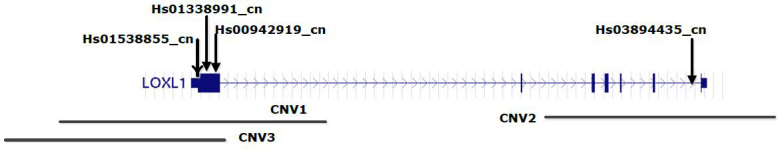
The relative location of selected copy number real-time PCR assays and identified CNVs from the array CGH for the *LOXL1* gene. The actual copy number assay ID was included in the figure. Three assays (Hs01538855_cn, Hs01338991_cn, and Hs00942919_cn) are located in the exon 1 region while one assay (Hs03894435_cn) is located in intron 6 of the *LOXL1* gene. CNV1, CNV2, and CNV3 match CNV #1, #2, and #3, respectively, in Table 1.

We designed a custom array CGH based on the CGH 4X72k array from Roche NimbleGen. The size of the area examined in the *LOXL1* genomic region was 1.5 Mb (chr15:71,250,002–72,749,772, Hg18) and was centered on the *LOXL1* gene. More than 42,000 probes were used in the analysis (Figure 1). These designed probes ranged from 50 to 72 bp to optimize their thermodynamic properties. The mean interval between adjacent probes was 27 bp. No probes were designed within genomic regions with repeated sequences, represented by the gaps in Figure 1. After quality control and data analysis, we identified three potential DNA copy number variations in the *LOXL1* region (Table 1), which met the following criteria: 1) at least ten probes per candidate CNV, 2) genomic size ≥1 kb, and 3) good-quality metrics of array CGH hybridization. Two CNV candidates, CNV1 and CNV3, overlapped with exon 1 and intron 1 of the *LOXL1* gene while CNV2 overlapped exons 3–7 of the *LOXL1* gene (Figure 2).

**Table 1:** List of DNA copy number changes in exfoliation glaucoma cases from South Africa.

CNV #	Gain/Loss	Size (bp)	Start	Stop	# probes	# cases
1	Gain	9843	72,001,694	72,011,537	359	1
2	Gain	14,072	72,023,576	72,037,648	488	1
3	Gain	3139	72,004,249	72,007,388	116	1

bp is for base pair. The genomic boundaries were defined in reference to the NCBI36/hg18 human genome build. The DNA copy number variants were identified through array CGH (comparative genomic hybridization). The data analysis was done with NimbleScan software.

**Figure 3:**
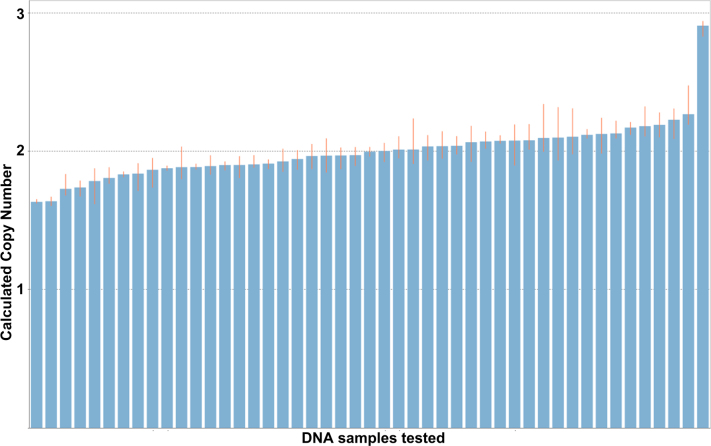
DNA copy number variations with DNA samples tested using Hs01538855_cn copy number assay from Applied Biosystems. Each DNA sample was run with four replicates with 10 ng DNA. A predesigned reference assay against the RNase P gene was included in the real-time PCR experiment for each sample. The data from real-time PCR was analyzed using CopyCaller v1.0 software from Applied Biosystems. All the CNV calls were generated with >95% confidence.

To validate the DNA copy number changes, we selected four predesigned DNA copy number assays, whose relative genomic locations are shown in Figure 2. The first three assays (Hs01538855_cn, Hs01338991_cn, and Hs00942919_cn) targeted the first exon and were used to validate CNV1 and CNV3 identified in the aCGH experiment. The assay Hs03894435_cn targeted intron 6 and was used to validate CNV2 identified in that experiment. TaqMan-based real-time PCR reactions in quadruplicate failed to validate any of the three identified DNA duplication events. Instead, we found that a single control sample had a DNA duplication (Figure 3). This DNA duplication was confirmed with all four copy number assays (data not shown), indicating that this specific DNA duplication in this unaffected individual covered the entire *LOXL1* genomic region. The frequency of this duplication was not significantly different between the XFG cases and the controls (Fisher’s exact test, two-tailed p value=0.36).

## Discussion

This study is the first study of *LOXL1* copy number variants in exfoliation glaucoma conducted in black South African subjects. We applied a comprehensive approach using array CGH and copy number real-time PCR assays. We failed to identify *LOXL1* CNVs that were significantly different between the XFG cases and the controls.

Although our initial CGH array analysis identified three potential CNVs in the XFG cases, these CNVs were not validated with TaqMan probe-based copy number real-time PCR assays. The lack of validation might be related to the DNA probes selected in the CGH array. Many of the probes on the CGH array overlapped since the median interval between adjacent probes was 27 bp and the length of the probes ranged from 50 bp to 72 bp. This probe overlap may have interfered with the DNA hybridization and led to false-positive CNV findings, which might be the main reason for our failure to validate the identified CNVs. The CNV-specific real-time PCR assays were performed with four replicates for every sample with an internal reference assay targeting the RNase P gene. This robust design enabled more reliable and replicable real-time PCR results. Therefore, validation of CNV-specific real-time PCR was considered the main criterion in our study. This study underscores the importance of validation when studying CNVs. The array CGH covers a relatively large genomic region (>1 kb at least) while real-time PCR-based CNV assays target a relatively small genomic region (<100 bp).

The black South African subjects in this study are uniquely valuable and confer extraordinary power for studying XFG [15,16]. We have reported a strong association (p=5.2 × 10^−13^) between XFG disease status and the A allele of the variant rs3825942 despite a small data set [15,16]. This study examined the potential role of *LOXL1* copy number variants in this special population. Our study indicated a limited contribution of the potential common *LOXL1* copy number variants to XFG in black South African individuals. It will be necessary to replicate our negative findings in a larger sample of black South Africans and other populations.

This study was limited by the relatively small sample size. The 20 selected cases in the array CGH experiment were used to identify common CNVs (≥2.5% of allele frequency) in the *LOXL1* genomic region. Our approach may have missed potential CNVs with lower allele frequency. Our real-time PCR-based experimental finding was also limited by the relatively small number of cases and controls from South Africa. Previously, we reported the significant genetic risk associated with *LOXL1* coding variants rs1048661 and rs3825942 in the black South African population with odds ratio 23.2 and 0.092, respectively [16]. Assuming an CNV allele frequency of 0.05, we calculated using Quanto software [35] that our case-control data set has more than 99% power to detect either risk. More XFG cases and controls from South Africa could be integrated to improve the statistical power in the future.

In summary, we conducted a two-stage DNA copy number study in the *LOXL1* genomic region with XFG in a black South African population. We did not detect a significant contribution of common CNV in *LOXL1* to the increased risk of XFG. This study does not rule out the possible contribution of CNVs in other populations with XFS or XFG.

## References

[1] Wiggs JL (2007). Genetic etiologies of glaucoma.. Arch Ophthalmol.

[2] Quigley HA, Broman AT (2006). The number of people with glaucoma worldwide in 2010 and 2020.. Br J Ophthalmol.

[3] Liu Y, Allingham RR. Genetics of Glaucoma. Encyclopeida of Life Sciences (ELS): John Wiley & Sons 2010

[4] Liu Y, Allingham RR (2011). Molecular genetics in glaucoma.. Exp Eye Res.

[5] Ritch R (2008). The management of exfoliative glaucoma.. Prog Brain Res.

[6] Schlötzer-Schrehardt U, Naumann GO (2006). Ocular and systemic pseudoexfoliation syndrome.. Am J Ophthalmol.

[7] Ritch R, Schlotzer-Schrehardt U (2001). Exfoliation (pseudoexfoliation) syndrome: toward a new understanding. Proceedings of the First International Think Tank.. Acta Ophthalmol Scand.

[8] Schlötzer-Schrehardt U (2009). Molecular pathology of pseudoexfoliation syndrome/glaucoma–new insights from LOXL1 gene associations.. Exp Eye Res.

[9] Ntim-Amponsah CT, Amoaku WM, Ofosu-Amaah S, Ewusi RK, Idirisuriya-Khair R, Nyatepe-Coo E, Adu-Darko M (2004). Prevalence of glaucoma in an African population.. Eye (Lond).

[10] Cashwell LF, Shields MB (1988). Exfoliation syndrome. Prevalence in a southeastern United States population.. Arch Ophthalmol.

[11] Ball SF (1988). Exfoliation syndrome prevalence in the glaucoma population of South Louisiana.. Acta Ophthalmol Suppl.

[12] Rotchford AP, Kirwan JF, Muller MA, Johnson GJ, Roux P (2003). Temba glaucoma study: a population-based cross-sectional survey in urban South Africa.. Ophthalmology.

[13] Rotchford AP, Johnson GJ (2002). Glaucoma in Zulus: a population-based cross-sectional survey in a rural district in South Africa.. Arch Ophthalmol.

[14] Tishkoff SA, Reed FA, Friedlaender FR, Ehret C, Ranciaro A, Froment A, Hirbo JB, Awomoyi AA, Bodo JM, Doumbo O, Ibrahim M, Juma AT, Kotze MJ, Lema G, Moore JH, Mortensen H, Nyambo TB, Omar SA, Powell K, Pretorius GS, Smith MW, Thera MA, Wambebe C, Weber JL, Williams SM (2009). The genetic structure and history of Africans and African Americans.. Science.

[15] Rautenbach RM, Bardien S, Harvey J, Ziskind A (2011). An investigation into LOXL1 variants in black South African individuals with exfoliation syndrome.. Arch Ophthalmol.

[16] Williams SE, Whigham BT, Liu Y, Carmichael TR, Qin X, Schmidt S, Ramsay M, Hauser MA, Allingham RR (2010). Major LOXL1 risk allele is reversed in exfoliation glaucoma in a black South African population.. Mol Vis.

[17] Chen H, Chen LJ, Zhang M, Gong W, Tam PO, Lam DS, Pang CP (2010). Ethnicity-based subgroup meta-analysis of the association of LOXL1 polymorphisms with glaucoma.. Mol Vis.

[18] Thorleifsson G, Magnusson KP, Sulem P, Walters GB, Gudbjartsson DF, Stefansson H, Jonsson T, Jonasdottir A, Stefansdottir G, Masson G, Hardarson GA, Petursson H, Arnarsson A, Motallebipour M, Wallerman O, Wadelius C, Gulcher JR, Thorsteinsdottir U, Kong A, Jonasson F, Stefansson K (2007). Common sequence variants in the LOXL1 gene confer susceptibility to exfoliation glaucoma.. Science.

[19] Fingert JH, Alward WL, Kwon YH, Wang K, Streb LM, Sheffield VC, Stone EM (2007). LOXL1 mutations are associated with exfoliation syndrome in patients from the midwestern United States.. Am J Ophthalmol.

[20] Hewitt AW, Sharma S, Burdon KP, Wang JJ, Baird PN, Dimasi DP, Mackey DA, Mitchell P, Craig JE (2008). Ancestral LOXL1 variants are associated with pseudoexfoliation in Caucasian Australians but with markedly lower penetrance than in Nordic people.. Hum Mol Genet.

[21] Pasutto F, Krumbiegel M, Mardin CY, Paoli D, Lammer R, Weber BH, Kruse FE, Schlotzer-Schrehardt U, Reis A (2008). Association of LOXL1 common sequence variants in German and Italian patients with pseudoexfoliation syndrome and pseudoexfoliation glaucoma.. Invest Ophthalmol Vis Sci.

[22] Kim S, Kim Y (2012). Variations in LOXL1 associated with exfoliation glaucoma do not affect amine oxidase activity.. Mol Vis.

[23] Lee RK (2008). The molecular pathophysiology of pseudoexfoliation glaucoma.. Curr Opin Ophthalmol.

[24] Zhang F, Gu W, Hurles ME, Lupski JR (2009). Copy number variation in human health, disease, and evolution.. Annu Rev Genomics Hum Genet.

[25] Liu Y, Gibson J, Wheeler J, Kwee LC, Santiago-Turla CM, Akafo SK, Lichter PR, Gaasterland DE, Moroi SE, Challa P, Herndon LW, Girkin CA, Budenz DL, Richards JE, Allingham RR, Hauser MA (2011). GALC deletions increase the risk of primary open-angle glaucoma: the role of Mendelian variants in complex disease.. PLoS ONE.

[26] Davis LK, Meyer KJ, Schindler EI, Beck JS, Rudd DS, Grundstad AJ, Scheetz TE, Braun TA, Fingert JH, Alward WL, Kwon YH, Folk JC, Russell SR, Wassink TH, Sheffield VC, Stone EM (2011). Copy number variations and primary open-angle glaucoma.. Invest Ophthalmol Vis Sci.

[27] Fingert JH, Robin AL, Stone JL, Roos BR, Davis LK, Scheetz TE, Bennett SR, Wassink TH, Kwon YH, Alward WL, Mullins RF, Sheffield VC, Stone EM (2011). Copy number variations on chromosome 12q14 in patients with normal tension glaucoma.. Hum Mol Genet.

[28] Meyer KJ, Davis LK, Schindler EI, Beck JS, Rudd DS, Grundstad AJ, Scheetz TE, Braun TA, Fingert JH, Alward WL, Kwon YH, Folk JC, Russell SR, Wassink TH, Stone EM, Sheffield VC (2011). Genome-wide analysis of copy number variants in age-related macular degeneration.. Hum Genet.

[29] Kubista KE, Tosakulwong N, Wu Y, Ryu E, Roeder JL, Hecker LA, Baratz KH, Brown WL, Edwards AO (2011). Copy number variation in the complement factor H-related genes and age-related macular degeneration.. Mol Vis.

[30] Liu MM, Agron E, Chew E, Meyerle C, Ferris FL, Chan CC, Tuo J (2011). Copy number variations in candidate genes in neovascular age-related macular degeneration.. Invest Ophthalmol Vis Sci.

[31] Sawitzke J, Im KM, Kostiha B, Dean M, Gold B (2011). Association assessment of copy number polymorphism and risk of age-related macular degeneration.. Ophthalmology.

[32] Khan TT, Li G, Navarro ID, Kastury RD, Zeil CJ, Semchyshyn TM, Moya FJ, Epstein DL, Gonzalez P, Challa P (2010). LOXL1 expression in lens capsule tissue specimens from individuals with pseudoexfoliation syndrome and glaucoma.. Mol Vis.

[33] Schlötzer-Schrehardt U, Pasutto F, Sommer P, Hornstra I, Kruse FE, Naumann GO, Reis A, Zenkel M (2008). Genotype-correlated expression of lysyl oxidase-like 1 in ocular tissues of patients with pseudoexfoliation syndrome/glaucoma and normal patients.. Am J Pathol.

[34] Miller SA, Dykes DD, Polesky HF (1988). A simple salting out procedure for extracting DNA from human nucleated cells.. Nucleic Acids Res.

[35] Gauderman WJ (2002). Sample size requirements for matched case-control studies of gene-environment interaction.. Stat Med.

